# The effect of sodium nitroprusside on cerebral haemodynamic and headache in healthy subjects

**DOI:** 10.1186/1129-2377-1-S14-P232

**Published:** 2013-02-21

**Authors:** S Guo, J Olesen, M Ashina, S Birk

**Affiliations:** 1 Danish Headache Center, Department of Neurology, Glostrup Hospital, Denmark; 2 Headache Center and Department of Neurology, Glostrup Hospital, Faculty of Health Sciences, Universi, Denmark; 3 Department of Clinical Neurophysiology, Rigshospitalet, Faculty of Health Sciences, University of Copenhagen, Denmark

## 

Sodium nitroprusside (SNP) is a powerful vasodilatatory agent, which similar to GTN releases nitric oxide (NO) but in contrast does not to pass blood-brain barrier. Nevertheless it has already been used in animal models without any knowledge of its headache inducing potential. We hypothesized that SNP would induce headache and vasodilation of cephalic and radial but not cerebral arteries. Five healthy volunteers received intravenous infusion of SNP in a non-randomized dose-titration (1-5 µg/kg/min) study. We recorded headache intensity (verbal rating scale from 0–10), velocity in the middle cerebral artery (VMCA), and diameters of the superficial temporal artery (STA) and radial artery (RA). All participants reported a dose related headache (median peak = 2.5, range 0-3). SNP dilated the STA and RA, caused a marked increase of heart rate and a decrease of mean arterial pressure (MAP) and PetCO2. We found that SNP decreased the velocity of the MCA, but this was cancelled by a decrease of CBF due to hypocapnia. In conclusion, the present study shows that SNP is a headache inducing agent with close similarities to headaches induced by GTN and possibly without effect on intracerebral arteries.

